# Radiological Comparison of Canal Fill between Collared and Non-Collared Femoral Stems: A Two-Year Follow-Up after Total Hip Arthroplasty

**DOI:** 10.3390/jimaging10050099

**Published:** 2024-04-25

**Authors:** Itay Ashkenazi, Amit Benady, Shlomi Ben Zaken, Shai Factor, Mohamed Abadi, Ittai Shichman, Samuel Morgan, Aviram Gold, Nimrod Snir, Yaniv Warschawski

**Affiliations:** Division of Orthopedics, Tel Aviv Sourasky Medical Center, Faculty of Medicine, Tel Aviv University, Tel Aviv 6423906, Israel

**Keywords:** total hip arthroplasty (THA), stem–canal fill ratio (CFR), collared vs. non-collared, fixation indicators, radiological assessment

## Abstract

Collared femoral stems in total hip arthroplasty (THA) offer reduced subsidence and periprosthetic fractures but raise concerns about fit accuracy and stem sizing. This study compares collared and non-collared stems to assess the stem–canal fill ratio (CFR) and fixation indicators, aiming to guide implant selection and enhance THA outcomes. This retrospective single-center study examined primary THA patients who received Corail cementless stems between August 2015 and October 2020, with a minimum of two years of radiological follow-up. The study compared preoperative bone quality assessments, including the Dorr classification, the canal flare index (CFI), the morphological cortical index (MCI), and the canal bone ratio (CBR), as well as postoperative radiographic evaluations, such as the CFR and component fixation, between patients who received a collared or a non-collared femoral stem. The study analyzed 202 THAs, with 103 in the collared cohort and 99 in the non-collared cohort. Patients’ demographics showed differences in age (*p* = 0.02) and ASA classification (*p* = 0.01) but similar preoperative bone quality between groups, as suggested by the Dorr classification (*p* = 0.15), CFI (*p* = 0.12), MCI (*p* = 0.26), and CBR (*p* = 0.50). At the two-year follow-up, femoral stem CFRs (*p* = 0.59 and *p* = 0.27) were comparable between collared and non-collared cohorts. Subsidence rates were almost doubled for non-collared patients (19.2 vs. 11.7%, *p* = 0.17), however, not to a level of clinical significance. The findings of this study show that both collared and non-collared Corail stems produce comparable outcomes in terms of the CFR and radiographic indicators for stem fixation. These findings reduce concerns about stem under-sizing and micro-motion in collared stems. While this study provides insights into the collar design debate in THA, further research remains necessary.

## 1. Introduction

Total hip arthroplasty (THA) is a reconstructive orthopedic surgical procedure that involves replacing the damaged hip joint with an artificial prosthetic implant. It represents the gold-standard treatment for end-stage hip osteoarthritis, inflammatory arthritis, fractures, osteonecrosis, and other debilitating conditions of the hip joint when conservative management has failed [1,2]. In 2019, the annual volume of primary THA in the United States was 480,958, and the number of revision THAs was 30,541. Shichman et al. reported on the epidemiology of THA in the United States, projecting that the annual volume of primary THA would grow 139% by 2040, reaching 719,362 cases, and that revision THAs are projected to grow by 4.67%, reaching 43,514 cases [3,4]. The primary objectives of THA are to relieve intractable pain, restore mobility and function, improve quality of life, and correct deformities of the affected hip joint. During the procedure, the diseased or damaged portions of the hip, including the femoral head and acetabulum, are surgically removed and replaced with prosthetic components made of metal, ceramic, or polyethylene bearings [5]. THA is one of the most performed and successful orthopedic operations globally. Its utilization has increased dramatically over the past few decades due to the aging population, improved implant designs/materials, advancements in surgical techniques, and the expansion of indications [6]. Numerous studies have demonstrated the excellent long-term durability, implant survivorship, and favorable clinical outcomes of modern THA [7]. Factors contributing to the success of THA include appropriate patient selection, meticulous surgical techniques adhering to principles of biomechanics, optimized implant sizing/positioning, bone preservation, accelerated rehabilitation protocols, and the prevention of complications like instability, infection, and thromboembolic events [8,9,10]. Continued research into implant designs, bearing surfaces, fixation methods, and novel technologies further improves outcomes and longevity [11].

Uncemented stems in THA have become the dominant fixation strategy for individuals under the age of 75 due to their simple implantation, excellent stability, favorable clinical outcomes, and robust implant survival rates [11,12,13]. Nevertheless, apprehensions persist regarding the accuracy of the fit of uncemented stems within the proximal femoral canal, the reproducibility of the final seating height of the implant, and the likelihood of stem subsidence [14,15]. An inadequate match between the implant and bone geometry may lead to distal wedging, insufficient proximal contact, and micro-motion that predisposes to radiolucency formation [16]. Therefore, selecting an appropriate stem size is paramount to attain optimal stress distribution on the proximal femur and achieve biological fixation [17]. However, significant individual anatomical variations in the morphology of the proximal femur can give rise to inaccuracies in the determination of the implant size, which may subsequently culminate in the need for early revision surgery [18,19].

The utilization of uncemented, non-collared femoral stems represent a dependable and frequently employed procedure in THA. The insertion of an uncemented stem necessitates sufficient press fit to establish immediate stability of the implant. Immediate stability serves as the foundation for subsequent ingrowth and integration processes. Throughout this phase, the stem is susceptible to both vertical and rotational subsidence, posing a risk of secondary integration in an unintended position. Should subsidence occur, it may adversely affect functional outcomes. Moreover, a subsiding stem is predisposed to inadequate secondary integration, potentially leading to micro-motion and partial fibrous fixation. Calcar collars partially addresses these issues, as collared stems are designed to facilitate the transfer of loads to the excised femoral calcar and prevent implant subsidence within the cancellous bone of the metaphysis [20,21]. This is supported by several studies showing that the use of collars could improve stem survival and facilitate revision THA in cases of massive femoral bone defects [20,22]. Furthermore, the utilization of a collared stem has been reported to reduce stem subsidence and rotation, as well as early periprosthetic fractures [23,24]. However, the utility of collars has remained controversial since their development, with inconclusive evidence regarding their advantages in both short- and long-term clinical results when contrasted with non-collared stems [25,26]. Moreover, there have been concerns regarding the potential of under-sizing of the femoral stem while relying on the collar for stability [27], which can potentially lead to stem micro-motion and loosening [16]. Nevertheless, currently, there is a lack of evidence assessing the association between the utilization of a collared stem and proper stem sizing.

Therefore, the main objective of this study is to compare the stem–canal fill ratio (CFR), used as a surrogate for proper stem sizing, between the collared and non-collared cementless stems of similar design. The secondary objective is to compare radiographic parameters indicative of stem fixation. Our hypothesis postulates that using collared stems will lead to a reduced CFR. The results of this study may offer guidance to surgeons in selecting implants for their patients and potentially enhance the long-term outcomes of THA.

## 2. Methods

### 2.1. Study Design

This retrospective, single-center study analyzed a consecutive cohort of patients who underwent a cementless primary THA between August 2015 and October 2020 utilizing the Corail cementless stem Total Hip System (Depuy, Warsaw, IN, USA). Patients were stratified into two groups based on whether they received a collared or a non-collared stem. Patients with clinical and radiographic follow-ups of less than two years were excluded from analysis. The decision to focus exclusively on data from the same implant aimed to reduce potential confounding variables. The data point of two years following surgery was chosen because previous reports have shown that periprosthetic radiolucency, bone remodeling, and component migration generally occur within the first two years following surgery [28].

### 2.2. Baseline Demographics and Stratification

The baseline demographic characteristics of the patients were obtained from electronic medical records. These characteristics included age, body mass index (BMI), gender, and American Society of Anesthesiologists (ASA) classification [29]. Age was recorded as the patient’s chronological age in years at the time of surgery. It is an important factor to consider, as bone quality and healing potential can decline with older age. Higher BMI values indicate greater adiposity. BMI is relevant, as obesity has been associated with increased surgical risks and poorer outcomes in joint arthroplasty. The American Society of Anesthesiologists (ASA) physical status classification system assesses a patient’s preoperative medical comorbidities. The ASA scores range from I (normal healthy patient) to VI (declared brain dead). Higher ASA classes indicate greater severity of systemic disease and anesthetic risk. Preoperative comorbidities may impact surgical decisions, perioperative management, and the postoperative recovery course.

### 2.3. Preoperative Radiographic Assessment

**Figure 1:**
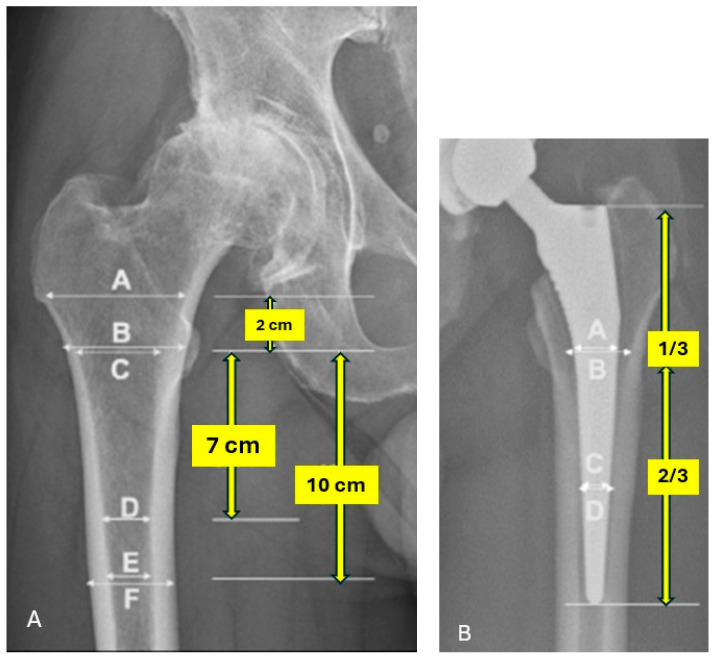
(**A**) Landmarks and guidelines for radiographic assessment preoperatively and (**B**) at latest follow-up.

Preoperative radiographic assessments were conducted using patients’ preoperative anterior–posterior hip radiographs and were aimed at assessing patients’ bone quality prior to surgery. The canal calcar ratio (CCR) is a measure of the femoral canal width relative to the calcar region and was determined by dividing the diameter of the femoral canal 10 cm distal to the lesser trochanter (E) by the diameter of the femoral canal at the midpoint of the lesser trochanter (C) (E/C, per Figure 1A) [30]. Higher CCR values indicate a wider femoral canal and potential poor bone quality. The canal flare index (CFI) quantifies the degree of femoral canal flaring and was calculated by dividing the femoral canal diameter 2 cm proximal to the lesser trochanter (A) by the femoral canal diameter 10 cm distal to the lesser trochanter (E) (A/E, per Figure 1A). Higher CFI values correspond to increased femoral canal flaring [31].

**Figure 2:**
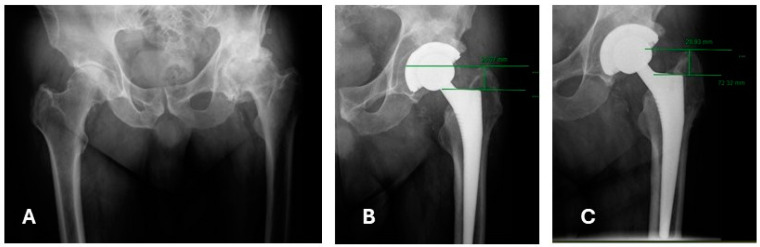
An AP X-ray of a 63 y/o patient (**A**) who underwent THA with a collarless stem due to osteoarthritis (**B**) and had a 3 mm stem subsidence 2 years postoperative (**C**).

The morphological cortical index (MCI) provides an assessment of cortical bone mass and was determined by calculating the ratio of the outer diameter of the femur at the level of the lesser trochanter (B) to the diameter of the femoral canal 7 cm distal to the lesser trochanter (D) (B/D, per Figure 1A) [31]. Lower MCI values indicate reduced cortical thickness and decreased bone mass. The canal bone ratio (CBR) measures the canal width relative to the outer femoral diameter and was calculated by dividing the diameter of the femoral canal 10 cm below the lesser trochanter (E) by the outer diameter of the femur 10 cm below the level of the lesser trochanter (F) (E/F, per Figure 1A) [32]. Higher CBR ratios signify a wider femoral canal in relation to the outer diameter (Figure 2).

The Dorr classification provides a qualitative assessment of femoral morphology and was assigned based on the CCR value (Type A, CCR < 0.5 indicating a wide metaphyseal canal; Type B, 0.5 < CCR < 0.75 indicating a normal metaphyseal canal; Type C, CCR > 0.75 indicating a narrow or stovepipe canal). This classification system helps guide implant selection and the surgical approach [31,33].

### 2.4. Postoperative Radiographic Assessment

Postoperative radiographic assessments were conducted using the patient’s most recent anterior–posterior hip radiographs, with images taken only at least two years after the surgical procedure being included for evaluation (Figure 1B). The length of the stem was assessed radiographically by measuring along the vertical axis, with the middle third of the stem being identified as starting one-third of the total stem length distal to the top of the stem and the distal third of the stem being identified as starting two-thirds of the total stem length distal to the top of the stem. At each point, the femoral stem–canal fill was calculated as the ratio of the stem width to the femoral canal diameter [34].

Adequate femoral component fixation was determined by the absence of radiolucent lines around its intramedullary surface and the presence of spot welds. Radiolucent lines surrounding the implant on radiographs indicate gaps or fibrous tissue at the bone–implant interface, suggesting impaired osseointegration and fixation. Spot welds refer to areas of endosteal bone formation directly onto the implant surface, providing mechanical interlock and biological fixation. Adequate femoral component stability was defined by the absence of pedestals below the tip of the femoral component, calcar atrophy, radiolucent lines, and particle shedding [33]. Pedestals are circumferential radiolucencies below the stem tip, signifying distal stem loosening. Calcar atrophy indicates excessive loading and prediction of future loosening. Radiolucent lines and particle shedding also indicate component instability and wear. The presence of subsidence was evaluated by measuring the change in distance from the top of the greater trochanter to the top of the stem, with positive stem subsidence defined as a change greater than or equal to 5 mm [35]. Excessive subsidence implies the incomplete transfer of load to the diaphysis and instability at the bone–implant interface. The radiographic analysis was conducted on the Picture Archiving and Communication System (PACS) using Visage 7 Imaging Software (Visage Imaging Inc., San Diego, CA, USA, Version 7.1.12e). The PACS enables the retrieval and viewing of medical images, while the dedicated software allows precise measurements on calibrated images. All radiographs were reviewed by one of two fellowship-trained surgeons from the author group (I.S. and Y.W.) to ensure consistent and expert evaluation. Interobserver reliability was tested using the intraclass correlation coefficient (ICC) with a 2-way random effect model, assuming single measurement and absolute agreement. The ICC quantifies the degree of agreement between multiple raters on the same set of subjects, accounting for potential systematic bias. The specific model used is appropriate for assessing the reliability of ratings from the two surgeons. The sample size for the reliability test was calculated with an ICC target value of 0.8 and a 95% confidence interval width of 0.2. A minimum number of interobserver reliability for the two raters was 20 by Bonnett’s approximation [36]. Thus, a subset of 20 radiographs was read by both the surgeons. The intraclass correlation coefficient (ICC) for measurements of canal morphology was 0.84 (0.79–0.90) and was 0.82 (0.77–0.89) for canal stem fill.

### 2.5. Statistical Analysis

First, a Shapiro–Wilk normality test was conducted on all the relevant data. Categorical variables were compared using the Chi-squared test or Fisher’s exact test, while continuous variables were compared using the independent-samples *t*-test. Statistical significance was set at a *p*-value < 0.05. Categorical variables are expressed as count (percentage), while continuous variables are expressed as mean ± standard deviation for parameters distributed normally and as median and interquartile ranges for parameters with a non-normal distribution. A statistical analysis was performed using IBM SPSS statistics (version 27.0.1).

## 3. Results

### 3.1. Patient Demographics and Preoperative Radiographic Assessment

A total of 202 THAs met our inclusion criteria. Of those, 103 (50.4%) were in the collared cohort, and 99 (49.6%) were in the non-collared cohort. While patients in the collared group were older (66.4 versus 61.7 years, *p* = 0.02) and were more likely to be ASA 2/3 (98 versus 88.9%, *p* = 0.01) when compared to the non-collared group, patients’ bone quality, as assessed by the CCR (*p* = 0.14), CFI (*p* = 0.12), MCI (*p* = 0.26), and CBR (*p* = 0.50), was similar between the cohorts. Similarly, the distribution of the Dorr classification was comparable between the cohorts (*p* = 0.15). Body mass index (BMI) did not significantly differ between the groups (*p* = 0.55), indicating comparable preoperative BMI distributions. Regarding primary diagnoses, no statistically significant differences were found between groups (*p* = 0.23), with osteoarthritis (OA) being the predominant diagnosis overall. These findings suggest that while demographic characteristics and primary diagnoses may differ slightly between the groups, preoperative radiographic parameters remain largely consistent, supporting the comparability of the study cohorts in terms of anatomical and radiographic features prior to hip arthroplasty (Table 1).

### 3.2. Postoperative Radiographic Outcomes at Two-Year Follow-Up

The median femoral stem CFRs at the distal and the middle thirds of the stem were comparable between both cohorts (*p* = 0.27 and *p* = 0.59, respectively). Similarly, an analysis of spot welds, a measure of bone ingrowth and implant stability, demonstrated no significant difference between the collarless and collared groups (*p* = 0.35). Similarly, the incidence of the pedestal sign, indicative of implant loosening, was negligible in both groups, with no statistically significant difference noted (*p* = 0.49). An evaluation of stress shielding, a common concern following hip arthroplasty, showed comparable rates between the collarless and collared implants (*p* = 0.65), suggesting similar load-sharing characteristics. The rates of patients experiencing substantial stem subsidence showed a slight upward trend among non-collared patients, although this difference was not statistically significant (collared 11.7% versus non-collared 19%, *p* = 0.17) (Table 2).

## 4. Discussion

The current study compared postoperative radiographic and clinical outcomes between patients who underwent THA with either a collared or a non-collared Corail stem. The key findings of our study are as follows: (1) The postoperative canal fill ratios (CFRs) at the middle and distal thirds of the stem were similar regardless of stem design. (2) The presence of signs indicating stem fixation or lack thereof, such as spot welds, pedestal formation, and stress shielding, was found to be similar between the two groups. (3) The rate of patients experiencing substantial stem subsidence (≥5 mm) at two years postoperative showed a slight upward trend among the non-collared patients, although this difference was not statistically significant. These findings collectively suggest that both collarless and collared hip arthroplasty techniques result in comparable radiographic outcomes in terms of implant stability, fixation, and bone ingrowth. Therefore, the choice between collarless and collared implants may be based on other clinical factors, with radiographic outcomes not significantly differing between the two techniques.

Collared stems offer additional axial stability and reduce the risk of subsidence and periprosthetic fractures [23,24]. However, their utilization remains limited, with a recent review indicating that fewer than half of THA patients receive a collared stem [37]. This limited utilization is partly attributed to concerns regarding the under-sizing of the femoral stem while relying on the collar for stability. Under-sized stems may promote micro-motion, leading to inferior load distribution and delayed stem fixation [33]. However, the findings of this current study indicate that postoperative CFRs are consistent irrespective of whether the stem is collared or non-collared, thus refuting these concerns. This finding has significant implications for surgical decision making and implant selection. As apprehensions regarding collared stems diminish, there may be a shift toward greater consideration of collared stem options in clinical practice.

Our analyses also showed that indicators for stem fixation, such as spot welds, pedestal formation, and stress shielding were similar between the collared and non-collared groups, further refuting concerns over under-sized stems resulting in delayed stem fixation. These findings are similar to previous studies that have also reported comparable or improved outcomes in terms of stem fixation for collared versus non-collared stems [38,39]. In their finite element analysis, Watanabe et al. [38] measured the micro-motion at the stem–bone interface between collared and non-collared stems. Their results showed that collared stems demonstrated equal to improved stability when a gap was left between the collar and the calcar and when the collar was in contact with the calcar, respectively. In particular, collar contact was highly effective in suppressing micro-motion proximal to the stem. These findings suggest that the utilization of collared implants may not significantly impact stem fixation, highlighting its aforementioned advantages in terms of periprosthetic fractures and stem subsidence.

While the CFR and stem fixation indicators serve as important radiographic evaluations for THA outcomes, the assessment of stem subsidence is of paramount importance, as early stem migration is a predictive factor for aseptic loosening, which potentially leads to the need for a revision surgery [40]. Our data suggest a slight upward trend in the rate of patients experiencing stem subsidence among non-collared patients. Our findings align with previous studies, such as those conducted by Nerys-Figueroa et al. [24] and Giovanoulis et al. [37], which have contributed to our understanding of the association between the use of a collared stem and the reduction in the risk of stem subsidence.

Comparisons between stems with collars and those without collars have been extensively investigated in mechanical experiments utilizing cadaveric specimens and simulated bones, as well as in finite element analyses (FEA). It has been documented that stems with collars exhibit superior vertical and rotational stabilities compared to their collarless counterparts. Furthermore, mechanical studies have demonstrated that collared stems display greater resistance to periprosthetic fractures [23,41,42].

While the rate of subsidence in the non-collared group patients was almost twice as high as that in their collared counterparts, it is noteworthy that this difference was not statistically significant, most likely due to cohort size and lack of statistical power. Therefore, further research and larger-scale studies are essential to fully elucidate the clinical implications of these trends among non-collared implant patients.

Moreover, surgeons should explore other techniques to further improve surgical outcomes and reduce the complication rate, such as the use of new composite biocompatible materials and biocompatible coatings for prostheses [43,44,45].

Interestingly, the cohort of patients with collared implants was significantly older and had more comorbidities, as indicated by the ASA classification. Over the study period, surgeons at our institution gradually shifted from non-collared to collared stem implants. Therefore, it is possible that this observation stems from the temporal trends in the demographics of the hip fracture population, which have been previously reported to be aging and with increasing medical complexities. Holt et al. projected that by 2031, approximately 45% of hip fractures will occur in patients aged 85 years and above, compared to 34% in 2004 [46]. Similarly, Baker et al. demonstrated that the hip fracture population has increasingly complex medical, social, and rehabilitation care needs [47]. Despite these differences in patient demographics, the preoperative bone quality assessed by the Dorr classification was similar between the cohorts. Moreover, despite being older and having more comorbidities, patients with collared implants achieved comparable radiographic outcomes, further supporting the superiority of collared implants.

This study has several limitations. First, its retrospective nature introduces biases such as selection bias, missing data, and bias due to loss to follow-up. Additionally, being a single-center study focusing on a single implant may limit the generalizability of the findings to other facilities or implants. The most significant limitation is the limited sample size, with only 202 THAs included, which may have restricted the ability to detect smaller but clinically significant differences and limit generalizability to larger patient populations. Despite efforts to ensure consistency in radiographic assessments through interobserver reliability testing, variations between raters could still affect measurement accuracy. Moreover, the study may not have accounted for all potential confounding variables that could influence outcomes, such as patient comorbidities or surgical techniques beyond collar design. Future studies should address these limitations to provide more robust insights into the impact of collar design on THA outcomes.

## 5. Conclusions

The findings of this study indicate that both collared and non-collared Corail stems offer comparable outcomes in terms of the canal fill ratio and radiographic indicators for stem fixation. While collared stems provide additional axial stability and may reduce the risk of subsidence and periprosthetic fractures, concerns regarding under-sizing and micro-motion are refuted by our data. The observed trend of slight upward stem subsidence among non-collared patients warrants further investigation, and larger-scale studies are needed to fully understand the clinical implications of this trend and to guide surgical decision making. Overall, this study contributes to the ongoing discussion regarding the selection of collar design in THA and highlights the importance of evidence-based decision making and preoperative planning.

## Figures and Tables

**Table 1:** Patient demographics and preoperative radiographic assessment.

	Overall	Non-Collared	Collared	*p*-Value
Number of patients	202	99	103	
Sex (Men), *n* (%)	82 (41.6)	45 (45.5)	37 (35.9)	0.34
Mean Age (SD)	63.94 (13.9)	61.7 (15.2)	66.4 (12.1)	0.02
Mean BMI (SD)	28.7 (5)	29 (5.1)	28.6 (4.9)	0.55
ASA, *n* (%)				0.01
1	13 (6.6)	11 (11.1)	2 (2.0)	
2	144 (73.1)	64 (64.6)	80 (81.6)	
3	40 (20.3)	24 (24.2)	16 (16.3)	
Primary Dx., *n* (%)				0.23
AVN	17 (8.6)	8 (8.1)	9 (9.2)	
DDH	6 (3.0)	6 (6.1)	0 (0.0)	
OA	167 (84.8)	81 (81.8)	86 (87.8)	
AVN and OA	3 (1.5)	1 (1.0)	2 (2.0)	
Other	4 (2.0)	3 (3.0)	1 (1.0)	
Mean CCR (E/C) (SD) *	0.5 (0.1)	0.47 (0.8)	0.44 (0.4)	0.14
Mean CFI (A/E) (SD) *	3.4 (0.6)	3.4 (0.6)	3.5 (0.7)	0.12
Mean MCI (B/D) (SD) *	2.8 (0.4)	2.7 (0.4)	2.8 (0.4)	0.26
Mean CBR (E/F) (SD) *	0.5 (0.1)	0.5 (0.1)	0.5 (0.1)	0.50
Dorr Classification, *n* (%)				0.15
A	140 (69.3)	64 (64.6)	76 (73.8)	
B	62 (30.7)	35 (35.4)	27 (26.3)	
C	0 (0.0)	0 (0.0)	0 (0.0)	

SD, standard deviation; BMI, body mass index; ASA, American Society of Anesthesiologists; Dx, diagnosis; AVN, avascular necrosis; DDH, developmental dysplasia of the hip; OA, osteoarthritis; CCR, canal calcar ratio; CFI, canal flare index; MCI, morphological cortical index; CBR, canal bone ratio. * Ratios are based on Figure 1A.

**Table 2:** Postoperative radiographic assessment.

	Collared (*n* = 103)	Non-Collared (*n* = 99)	*p*-Value
Spot Welds, *n* (%)	20 (19.4)	14 (14.1)	0.35
Pedestal Sign, *n* (%)	0 (0.0)	1 (1.0)	0.49
Stress Shielding, *n* (%)	29 (28.2)	31 (31.3)	0.65
Subsidence ≥ 5 mm, *n* (%)	12 (11.7)	19 (19.2)	0.17
Median A/B Ratio [IQR] *	1.1 [1.06, 1.2]	1.1 [1.05, 1.18]	0.59
Median C/D Ratio [IQR] *	1.9 [1.8, 2.1]	1.9 [1.75, 2.16]	0.27

IQR, interquartile range. * Ratios are based on Figure 1B.

## Data Availability

Data are available upon reasonable request from the corresponding author.
